# Preliminary results from ASCENT-J02: a phase 1/2 study of sacituzumab govitecan in Japanese patients with advanced solid tumors

**DOI:** 10.1007/s10147-024-02589-x

**Published:** 2024-09-20

**Authors:** Yoichi Naito, Seigo Nakamura, Nobuko Kawaguchi-Sakita, Takanori Ishida, Takahiro Nakayama, Yutaka Yamamoto, Norikazu Masuda, Koji Matsumoto, Takahiro Kogawa, Kazuki Sudo, Akihiko Shimomura, Catherine Lai, Danjie Zhang, Yuki Iwahori, Dianna Gary, Danh Huynh, Hiroji Iwata

**Affiliations:** 1 Department of General Internal Medicine, National Cancer Center Hospital East, 6-5-1 Kashiwanoha, Kashiwa, Chiba 277-8577 Japan; 2 Showa University Hospital, Tokyo, Japan; 3 Kyoto University Hospital, Kyoto, Japan; 4 Tohoku University Hospital, Miyagi, Japan; 5 Osaka International Cancer Institute, Osaka, Japan; 6 Kumamoto University Hospital, Kumamoto, Japan; 7 Nagoya University Graduate School of Medicine, Nagoya, Japan; 8 Hyogo Cancer Center, Hyogo, Japan; 9 The Cancer Institute Hospital of JFCR, Tokyo, Japan; 10 National Cancer Center Hospital, Tokyo, Japan; 11 National Center for Global Health and Medicine, Tokyo, Japan; 12 Gilead Sciences, Inc., Foster City, CA USA; 13 Gilead Sciences, K.K., Tokyo, Japan; 14 Aichi Cancer Center Hospital, Aichi, Japan

**Keywords:** Metastatic triple-negative breast cancer, Sacituzumab govitecan, Antibody–drug conjugate, Phase 2, Japanese patients

## Abstract

**Background:**

Sacituzumab govitecan (SG) is a Trop-2–directed antibody–drug conjugate approved outside Japan for second-line and later metastatic triple-negative breast cancer (mTNBC), based on the ASCENT study (NCT02574455). We report SG safety and efficacy in an open-label, phase 1/2 bridging study in Japanese patients with advanced solid tumors (ASCENT-J02; NCT05101096; jRCT2031210346).

**Methods:**

Phase 1 was a standard 3 + 3 design. Patients received intravenous SG 6 mg/kg, escalating to 10 mg/kg, on Days 1 and 8 per 21-day cycle; primary endpoints were safety, incidence of dose-limiting toxicity/toxicities (DLTs), and determination of the recommended phase 2 dose (RP2D). In the multicohort phase 2 study, patients in the mTNBC cohort with previously treated disease received SG at the RP2D; primary endpoint was independent review committee (IRC)-assessed objective response rate (ORR; RECIST v1.1). Safety was a secondary endpoint.

**Results:**

In phase 1 (*N* = 15), one DLT (grade 3 elevated transaminases) occurred with SG 10 mg/kg; RP2D was SG 10 mg/kg regardless of *UGT1A1* status. In phase 2, 36 patients with mTNBC received SG 10 mg/kg. At median follow-up of 6.1 months, IRC-assessed ORR was 25.0% (95% CI 12.1–42.2; *P* = 0.0077). Median progression-free survival was 5.6 months (95% CI 3.9–not reached [NR]); median overall survival was NR. No treatment-emergent adverse events led to discontinuation or death.

**Conclusions:**

SG RP2D was established as 10 mg/kg in Japanese patients. SG showed efficacy in Japanese patients with previously treated mTNBC, a manageable safety profile, and no new safety signals, consistent with the previous ASCENT study.

**Supplementary Information:**

The online version contains supplementary material available at 10.1007/s10147-024-02589-x.

## Introduction

Triple-negative breast cancer (TNBC) is an aggressive subtype of breast cancer with few treatment options and poor outcomes [1]. TNBC is a heterogeneous disease, defined by the American Society of Clinical Oncology and the College of American Pathologists (ASCO/CAP) as estrogen/progesterone receptor (ER/PR) expression < 1% and human epidermal growth factor receptor 2 (HER2) immunohistochemistry (IHC) 0, 1 + , 2 + /in situ hybridization-negative (ISH −) [2, 3]. In Japan, breast cancer was the fourth leading cause of cancer-related deaths among women in 2021 [4]. TNBC accounts for ~ 15% of all breast cancers in Japan and is associated with a poor prognosis [5]. The standard of care in the first-line for patients with metastatic TNBC (mTNBC) is single-agent chemotherapy alone or in combination with immune checkpoint inhibitors in patients with programmed cell death-ligand 1 (PD-L1)-negative and PD-L1-positive (defined as immune cell [IC] score 1 +) TNBC, respectively. Other therapeutic options in the first-line include poly ADP-ribose polymerase (PARP) inhibitors in patients with germline *BRCA1* or *BRCA2* mutations [1, 6, 7]. However, beyond the first-line setting, there is no clear standard of care, including in Japan, with single-agent chemotherapy being frequently used [8, 9]. Chemotherapy is associated with low response rates, short progression-free survival (PFS), and significant toxicity among patients with pretreated mTNBC [1, 6, 10–13]. Consequently, there is a need for more effective treatment options for Japanese patients with mTNBC.

Sacituzumab govitecan (SG) is a trophoblast cell surface antigen 2 (Trop-2)–directed antibody–drug conjugate. SG is composed of a Trop-2 monoclonal antibody attached to SN-38, a potent topoisomerase I inhibitor, through a hydrolysable linker at a high drug-to-antibody ratio [14, 15]. SG delivers therapeutic concentrations of SN-38 to Trop-2–expressing tumor cells via rapid internalization and efficient release of the payload. Moreover, SN-38 can also diffuse out of the targeted cell and into the tumor microenvironment, resulting in the killing of adjacent tumor cells (“bystander” effect) [14, 15]. Trop-2 is highly expressed in all breast cancer subtypes and has been shown to be a viable target in TNBC [16, 17].

In the phase 3, global, randomized ASCENT study (IMMU-132–05; NCT02574455), SG demonstrated a significant survival benefit over treatment of physician’s choice chemotherapy with a manageable safety profile. In ASCENT, 529 patients with mTNBC that was relapsed or refractory to two or more previous chemotherapy regimens were enrolled [18]. In the SG versus chemotherapy groups, median PFS was 4.8 months versus 1.7 months (hazard ratio [HR], 0.43; 95% confidence interval [CI], 0.35–0.54), and median overall survival (OS) was 11.8 versus 6.9 months (HR, 0.51; 95% CI 0.41–0.62), respectively, in the full population, which included all randomly assigned patients including those with brain metastases. The incidence of adverse event (AE)-related discontinuations with SG treatment was low (5%), and clinically relevant grade 3 or 4 AEs such as neutropenia and diarrhea were managed using supportive medications and/or dose reductions [18]. Based on these results from ASCENT and findings from patients with mTNBC in the phase 1/2 basket study (IMMU-132–01; NCT01631552), SG has been approved in multiple countries, including in the United States (US), European Union, China, Singapore, and South Korea for the treatment of mTNBC after at least two prior systemic therapies (at least one in the metastatic setting) [18–23].

SG is also approved in the US and Europe for the treatment of hormone receptor (HR)-positive/HER2-negative (HR + /HER2 −) metastatic breast cancer (mBC) following endocrine-based therapy and at least two additional systemic therapies in the metastatic setting, based on results from the TROPiCS-02 study [23–25]. Furthermore, SG has received accelerated US Food and Drug Administration approval in the US for the treatment of locally advanced unresectable or metastatic urothelial cancer (mUC) following platinum-containing chemotherapy and either a programmed cell death 1 or PD-L1 inhibitor based on results from cohort 1 of the pivotal TROPHY-U-01 study [23, 26].

The ASCENT, TROPiCS-02, and TROPHY-U-01 studies were not conducted in Japan. There is a need to evaluate the safety, tolerability, and efficacy of SG in Japanese patients to confirm the results observed in studies in non-Japanese populations. ASCENT-J02 (NCT05101096; jRCT2031210346) is a phase 1/2 open-label study of SG in Japanese patients with advanced solid tumors. We report data from the phase 1 dose-finding portion of the study and from the cohort of patients with previously treated mTNBC in the phase 2 portion of the study.

## Patients and methods

### Study design and treatments

**Fig. 1:**
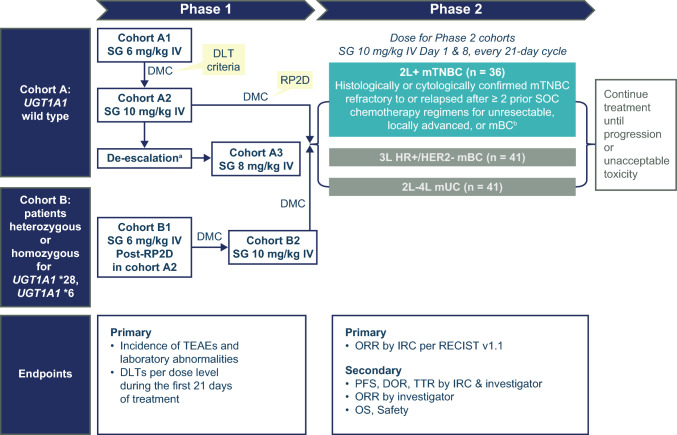
ASCENT-J02 study design. ^a^In the event that a DLT is observed in two out of six patients, enrollment of Cohort A3 at 8 mg/kg will begin; de-escalation to Cohort A3 was not needed.^b^Earlier adjuvant or neoadjuvant therapy for more limited disease will qualify as one of the required prior regimens if the development of unresectable, locally advanced, or metastatic disease occurred within 12 months after completion of chemotherapy. *2L* second-line, *3L* third-line, *4L* fourth-line, *DLT* dose-limiting toxicity, *DMC* data monitoring committee, *DOR* duration of response, *HR* + */HER2 − *hormone receptor-positive/human epidermal growth factor receptor 2-negative, *IRC* independent review committee, *IV* intravenous, *mBC* metastatic breast cancer, *mTNBC* metastatic triple-negative breast cancer, *mUC* metastatic urothelial cancer, *ORR* objective response rate, *OS* overall survival, *PFS* progression free survival, *RECIST v1.1* Response Evaluation Criteria in Solid Tumors v1.1, *RP2D* recommended phase 2 dose, *SG* sacituzumab govitecan*, SOC* standard of care, *TEAE* treatment-emergent adverse event, *TTR* time to response, *UGT1A1* UDP glucuronosyltransferase family 1 member A1

ASCENT-J02 is an open-label, multicenter, sequential dose-escalation and dose-expansion study evaluating the safety, tolerability, pharmacokinetics (PK), and efficacy of SG in Japanese patients. The study consists of 2 parts (Fig. 1). The phase 1 portion determined the recommended phase 2 dose of SG in Japanese patients with advanced solid tumors. Cohort A of the phase 1 portion enrolled patients who were wild type for the UDP glucuronosyltransferase family 1 member A1 (*UGT1A1*) genotype and used a standard 3 + 3 design, with a starting dose of SG 6 mg/kg and subsequent dose of SG 10 mg/kg, with de-escalation to SG 8 mg/kg as needed. The dose-limiting toxicity (DLT) period was 21 days. The safety and tolerability of each dose level was assessed by the data monitoring committee (DMC) after all patients in the cohort were followed for at least 21 days after the first dose of SG, or if patients had DLTs during the first 21 days of study drug dosing. Once the recommended phase 2 dose (RP2D) was determined in cohort A, enrollment of patients heterozygous or homozygous for the UGT1A1*28 and UGT1A1*6 alleles was to begin in cohort B. The phase 2 portion of ASCENT-J02 is a multicohort study of Japanese patients with mTNBC, HR + /HER2 − mBC, or mUC (Fig. 1). Patients in phase 2 were to receive SG at the RP2D.

In both phases, SG was administered as an intravenous (IV) infusion on Days 1 and 8 of continuous 21-day cycles. Patients continued to receive treatment until clinical or radiographic disease progression, unacceptable toxicity, or withdrawal of consent. Premedication and prophylaxis were permitted while on study treatment, and palliative and/or supportive medications (including granulocyte colony-stimulating factor [G-CSF]) were allowed at the investigator’s discretion.

### Patients

In phase 1, eligible patients had histologically or cytologically confirmed advanced solid tumors that were refractory or intolerant to all standard therapy. Cohort A of phase 1 comprised patients who were wild type for the *UGT1A1* genotype, and cohort B comprised patients who had a UGT1A1*28 or UGT1A1*6 polymorphism. In the phase 2 mTNBC cohort, eligible patients had histologically or cytologically confirmed TNBC that was refractory to or relapsed after at least two prior standard-of-care chemotherapy regimens for unresectable, locally advanced, or mBC; at least one of them for metastatic disease. Earlier adjuvant or neoadjuvant therapy for more limited disease qualified as one of the required prior regimens if the development of advanced disease occurred within 12 months after completion of chemotherapy. Patients in phase 2 had to have TNBC according to standard ASCO/CAP criteria (ER/PR < 1%; HER2 IHC 0, 1 + , 2 + /ISH −) [3]. All patients in both phases were also required to be ≥ 18 years of age, have an Eastern Cooperative Oncology Group performance status 0–1, and have adequate organ function. Patients with stable brain metastases for at least 4 weeks before treatment were eligible for the trial.

### Endpoints and assessments

In phase 1, the primary objective was to determine the RP2D and evaluate the safety and tolerability of SG. The primary endpoints were the incidence of treatment-emergent AEs (TEAEs), laboratory abnormalities, and DLTs per dose level during the first 21 days of treatment. Secondary endpoints included PK parameters for SG and free SN-38. In phase 2, the primary endpoint was the confirmed objective response rate (ORR) determined by the independent review committee (IRC) and defined as the proportion of patients who achieved a complete response (CR) or partial response (PR) confirmed after ≥ 4 weeks as assessed by Response Evaluation Criteria in Solid Tumors Version 1.1 (RECIST v1.1) in all treated patients [27]. Secondary endpoints in phase 2 included incidence of TEAEs and laboratory abnormalities, PFS (defined as the interval from the first dose of SG to the earlier of the first documentation of objective progressive disease [PD] or death from any cause) by investigator assessment and IRC, OS (defined as the time from date of first dose of SG to death from any cause), duration of response (DOR; defined as the time from the first documentation of CR or PR to the earlier of the first documentation of objective PD or death from any cause) by investigator assessment and IRC, time to response (TTR; defined as the time from the first dose of SG to the first documentation of CR or PR) by investigator assessment and IRC, and ORR by investigator assessment (Fig. 1).

Safety was evaluated in both phases in all treated patients until 30 days after the last dose of study treatment or initiation of alternative antitumor therapy, whichever occurred first. The severity of TEAEs was graded using the National Cancer Institute Common Terminology Criteria for Adverse Events v4.03. TEAEs were also assessed by causality/relationship to study drug. In phase 2, tumor assessment by computed tomography or magnetic resonance imaging was performed every 6 weeks for the first 24 weeks from Day 1 of Cycle 1, and every 9 weeks thereafter until disease progression requiring treatment discontinuation. Tumor response and progression were determined using RECIST v1.1.

### Statistical analysis

The safety and efficacy analysis sets comprised all patients who received at least one dose of study treatment. A total of up to 26 patients were planned to be enrolled in the phase 1 dose-escalation cohorts. In the phase 2 mTNBC cohort, a sample size of 35 patients was planned to be enrolled. The confirmed ORR by IRC was tested against the threshold of 10% using the exact binomial test at a one-sided significance level of 0.025. The confirmed ORR by IRC and the corresponding two-sided 95% CI based on the Clopper–Pearson method were determined along with the one-sided *P* value. The confirmed ORR by investigator and the corresponding two-sided 95% CI are provided. PFS, OS, and DOR were analyzed using the Kaplan–Meier method. TTR was summarized using descriptive statistics. The data being presented are from an early data cut at ~ 18 weeks after the last patient was enrolled in the phase 2 mTNBC cohort.

## Results

### Phase 1 dose escalation

#### Patient characteristics

**Fig. 2:**
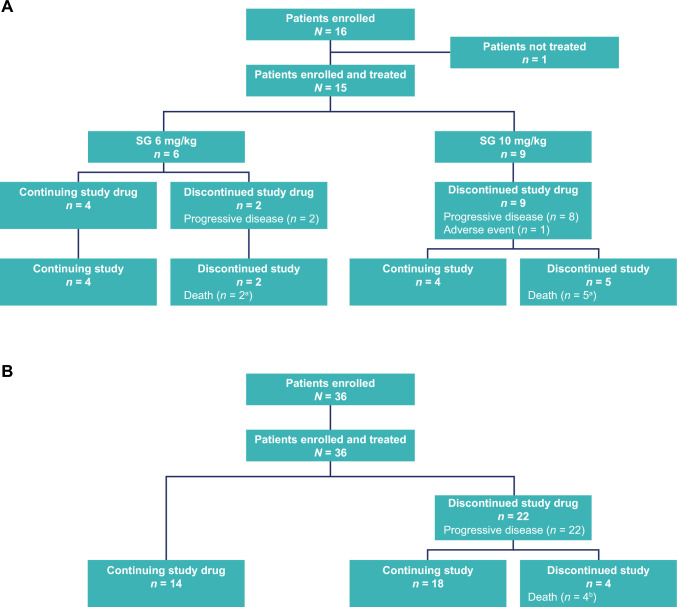
Patient disposition in **A** phase 1 and **B** phase 2 mTNBC of the ASCENT-J02 study. ^a^All deaths were due to disease progression and occurred more than 30 days after the last dose. ^b^All four deaths were due to disease progression; three deaths occurred more than 30 days after the last dose and one death occurred within 30 days of the last dose. *mTNBC* metastatic triple-negative breast cancer

Fifteen patients were treated with SG 6 mg/kg (*n* = 6) and 10 mg/kg (*n* = 9). Patients had a median age of 49 years, the majority had breast cancer (53%), and all patients had stage IV disease (Table 1). At the data cutoff date of May 12, 2023, two of six patients from the 6-mg/kg cohort and all patients from the 10-mg/kg cohort had discontinued the study drug (Fig. 2A). Among the patients treated with SG 10 mg/kg, six patients were wild type for the *UGT1A1* genotype, and three patients had a UGT1A1*28 or UGT1A1*6 polymorphism.

**Table 1:** Patient demographics and baseline characteristics in phase 1 dose-escalation cohorts

	SG 6 mg/kg (*n* = 6)	SG 10 mg/kg (*n* = 9)	Total (*N* = 15)
Sex at birth, *n* (%)			
Male	2 (33)	2 (22)	4 (27)
Female	4 (67)	7 (78)	11 (73)
Median age (range), years	51 (37–65)	49 (39–73)	49 (37–73)
Age group, *n* (%)			
< 65 years	5 (83)	7 (78)	12 (80)
≥ 65 years	1 (17)	2 (22)	3 (20)
Race or ethnic group, *n* (%)			
Japanese	6 (100)	9 (100)	15 (100)
ECOG PS, *n* (%)			
0	2 (33)	6 (67)	8 (53)
1	4 (67)	3 (33)	7 (47)
*UGT1A1* genotype, *n* (%)			
*1/*1 and G/G	2 (33)	6 (67)	8 (53)
*1/*1 and G/*6	2 (33)	2 (22)	4 (27)
*1/*28 and G/G	2 (33)	1 (11)	3 (20)
Cancer type, *n* (%)			
Breast	4 (67)	4 (44)	8 (53)
Head and neck	1 (17)	0	1 (7)
Pancreatic	1 (17)	0	1 (7)
Non-small cell lung	0	2 (22)	2 (13)
Ovarian	0	1 (11)	1 (7)
Colorectal	0	1 (11)	1 (7)
Esophageal	0	1 (11)	1 (7)
Cancer stage at screening, *n* (%)			
Stage IV	6 (100)	9 (100)	15 (100)

*ECOG PS* Eastern Cooperative Oncology Group performance status, *SG* sacituzumab govitecan, *UGT1A1* UDP glucuronosyltransferase family 1 member A1

#### Safety and RP2D

Any-grade TEAEs were observed in all patients treated, and 53% of patients had TEAEs of grade ≥ 3, all of which were deemed related to treatment (Table 2). The most frequent grade ≥ 3 TEAEs (≥ 15%) with SG 6 mg/kg was neutropenia (17%), and with SG 10 mg/kg were neutropenia (67%), leukopenia (33%), and anemia (22%). No DLTs were observed at SG 6 mg/kg and one DLT (grade 3 transaminases increased) was observed at 10 mg/kg (Table 2). There were no notable differences in TEAEs or safety outcomes by *UGT1A1* genotype (Supplementary Table 1; online only). In conjunction with a review and recommendation from the DMC, the RP2D of SG was established as 10 mg/kg, regardless of *UGT1A1* genotype.

**Table 2:** Safety summary in phase 1 dose-escalation cohorts

Safety-evaluable patients,^a^ *n* (%)	SG 6 mg/kg (*n* = 6)	SG 10 mg/kg (*n* = 9)	Total (*N* = 15)
Any-grade TEAEs	6 (100)	9 (100)	15 (100)
Related to study treatment	6 (100)	9 (100)	15 (100)
Grade ≥ 3 TEAEs	1 (17)	7 (78)	8 (53)
Related to study treatment	1 (17)	7 (78)	8 (53)
Serious TEAEs	0	1 (11)	1 (7)
Related to study treatment	0	0	0
TEAEs leading to temporary treatment interruption	3 (50)	7 (78)	10 (67)
TEAEs leading to dose reduction	1 (17)	2 (22)	3 (20)
TEAEs leading to treatment discontinuation	0	1 (11)	1 (7)
TEAEs leading to death	0	0	0
Dose-limiting toxicities^b^	0	1 (17)^c^	1 (11)

*AE* adverse event, *DLT* dose-limiting toxicity, *SG* sacituzumab govitecan, *TEAE* treatment-emergent adverse event

^a^TEAEs defined as any AEs that begin on or after the start of study drug through 30 days after the last dose of study drug or initiation of subsequent anti-cancer therapy, whichever occurs first

^b^DLT-evaluable patients (6 mg/kg [*n* = 3], 10 mg/kg [*n* = 6])

^c^Grade 3 transaminases increased

### Phase 2 dose expansion

#### Patient characteristics

In the phase 2 mTNBC cohort, 36 patients were enrolled and treated. At the data cutoff date of May 12, 2023, 22 patients had discontinued the study drug due to PD (Fig. 2B). Patients had a median age of 50 years, a majority of patients (72%) had an ECOG PS of 0, and most patients (94%) were aged < 65 years. A majority of patients (64%) had received two or three prior systemic anticancer regimens in the metastatic setting and 53% had received prior checkpoint inhibitor therapy (Table 3).

**Table 3:** Patient demographics and baseline characteristics in the phase 2 dose-expansion mTNBC cohort

	Phase 2 mTNBC cohort (*N* = 36)
Sex at birth, *n* (%)	
Male	0
Female	36 (100)
Median age (range), years	50 (29–73)
Age group, *n* (%)	
< 65 years	34 (94)
≥ 65 years	2 (6)
Race or ethnic group, *n* (%)	
Japanese	36 (100)
ECOG PS, *n* (%)	
0	26 (72)
1	10 (28)
Brain metastasis^a^	
Yes	3 (8)
No	33 (92)
Liver metastasis^a^	
Yes	12 (33)
No	24 (67)
Breast cancer stage at initial diagnosis	
Stage I–II	16 (44)
Stage III	14 (39)
Stage IV	5 (14)
Unknown	1 (3)
Breast cancer subtype at initial diagnosis	
TNBC	28 (78)
Non-TNBC	8 (22)
Prior systemic anticancer regimens for treatment of metastatic disease, *n* (%)	
1^b^	4 (11)
2 or 3	23 (64)
> 3	9 (25)
HER2 status, *n* (%)	
IHC 0	21 (58)
IHC 1 +	12 (33)
IHC 2 + /DISH − /FISH −	3 (8)
*UGT1A1* genotype, *n* (%)	
*1/*1 and G/G	32 (89)
*1/*1 and G/*6	2 (6)
*1/*28 and G/G	1 (3)
*28/*28 and G/G	1 (3)
Prior checkpoint inhibitor therapy, *n* (%)^c^	
Yes	19 (53)
No	17 (47)

*DISH* dual in situ hybridization, *ECOG PS* Eastern Cooperative Oncology Group performance status, *FISH* fluorescent in situ hybridization, *HER2* human epidermal growth factor receptor 2, *IHC* immunohistochemistry, *mTNBC* metastatic triple-negative breast cancer, *TNBC* triple-negative breast cancer, *UGT1A1* UDP glucuronosyltransferase family 1 member A1

^a^Per target/non-target lesions at screening

^b^Earlier adjuvant or neoadjuvant therapy for more limited disease will qualify as one of the required prior regimens if the development of unresectable, locally advanced, or metastatic disease occurred within 12 months after completion of chemotherapy

^c^Defined as cemiplimab, nivolumab, pembrolizumab, atezolizumab, avelumab, durvalumab, ipilimumab, tremelimumab, or spartalizumab; however, only nivolumab, pembrolizumab, and atezolizumab were administered

#### Efficacy outcomes

The median follow-up was 6.1 months (range 2.4–9.1). Confirmed ORR per IRC was 25% (95% CI 12.1–42.2; *P* = 0.0077) (Table 4). Best overall response by IRC was PR in 9 patients (25%), stable disease (SD) in 20 patients (56%), and PD in 6 patients (17%). ORR by investigator assessment was 28% (95% CI 14.2–45.2); 1 patient had CR (3%), 9 patients had PR (25%), 20 patients had SD (56%), and 6 patients had PD (17%). The median DOR per IRC was 6.2 months (95% CI 3.1–NR) and median TTR was 1.6 months (range,1.2–3.0) (Table 4). By investigator assessment, the median DOR and median TTR were 5.5 months (95% CI 3.9–NR) and 1.6 months (range 1.1–4.2), respectively.

**Table 4:** Summary of treatment efficacy in the phase 2 dose-expansion mTNBC cohort

	Independent review committee (*N* = 36)	Investigator assessment (*N* = 36)
Best overall response, *n* (%)		
Complete response	0	1 (3)
Partial response	9 (25)	9 (25)
Stable disease^a^	20 (56)	20 (56)
Progressive disease	6 (17)	6 (17)
Not evaluable	1 (3)	0
Confirmed ORR, %	25	28
95% CI	12.1–42.2	14.2–45.2
Duration of response, median (months)^b^	6.2^c^	5.5^d^
95% CI	3.1–NR	3.9–NR
Time to response, median (months)^e^	1.6^c^	1.6^d^
Range	1.2–3.0	1.1–4.2

Median follow-up for overall survival was 6.1 months, the primary analysis for efficacy was performed ~ 18 weeks after the last patient enrolled

*CI* confidence interval, *CR *complete response, *mTNBC* metastatic triple-negative breast cancer, *NR* not reached, *ORR* objective response rate, *PR* partial response

^a^Stable disease classified after a minimum duration of 6 weeks

^b^Duration of response (months) = (date of first event or censoring – date of first documented CR or PR + 1)/30.4357

^c^Evaluated in *n* = 9 patients

^d^Evaluated in *n* = 10 patients

^e^Time to response (months) = (date of first documented CR or PR – date of first dose + 1)/30.4375

**Fig. 3:**
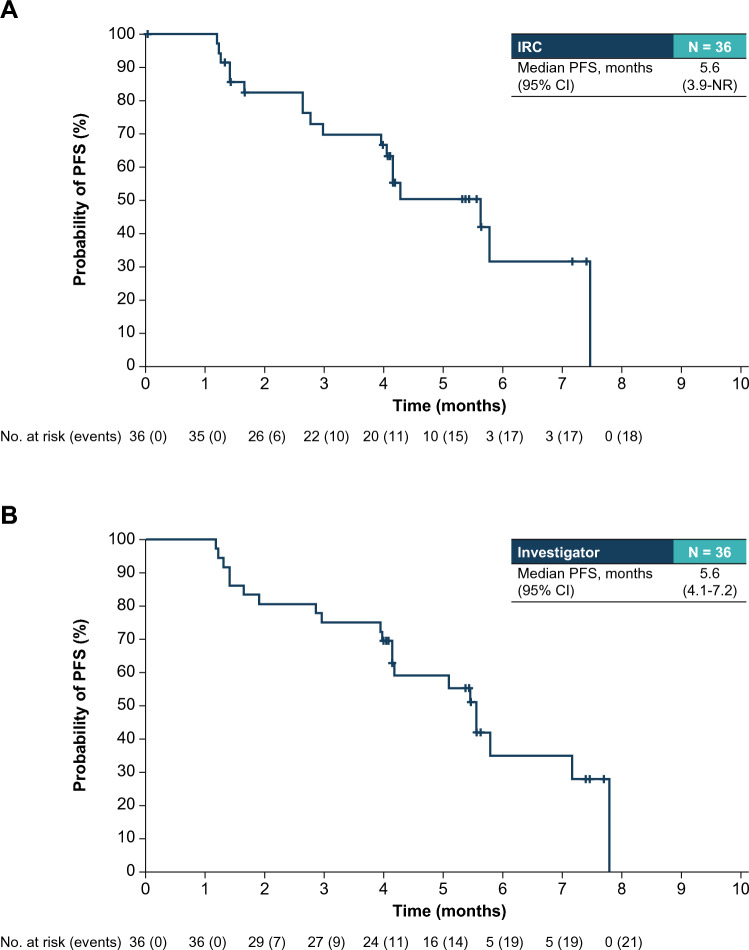
Kaplan–Meier estimate of PFS by **A** independent review committee and **B** by investigator assessment in the phase 2 dose-expansion mTNBC cohort of ASCENT-J02. Median follow-up for OS was 6.1 months, the primary analysis for efficacy was performed ~ 18 weeks after the last patient enrolled. *CI* confidence interval; *IRC* independent review committee; *mTNBC* metastatic triple-negative breast cancer; *NR* not reached; *PFS* progression-free survival

Median PFS was 5.6 months (95% CI 3.9–NR) by IRC (Fig. 3A) and 5.6 months (95% CI 4.1–7.2) by investigator assessment (Fig. 3B). Median OS was not reached at the time of this analysis (Supplementary Fig. 1; online only).

#### Safety

**Fig. 4:**
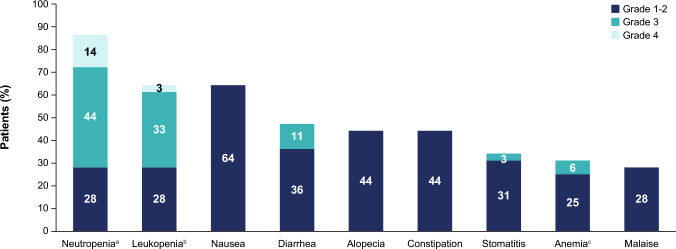
Any-grade TEAEs (≥ 25%) in the phase 2 dose-expansion mTNBC cohort (*N* = 36). ^a^Neutropenia includes neutrophil count decreased. ^b^Leukopenia includes white blood cell count decreased. ^c^Anemia includes hemoglobin decreased and red blood cell count decreased. *mTNBC* metastatic triple-negative breast cancer; *TEAE* treatment-emergent adverse event

The median duration of exposure to SG was 5.1 months (range 0.3–9.1) and the median number of treatment cycles received was 7 (range 1–13). TEAEs of grade ≥ 3 were observed in 72% of patients, all of which were assessed by the investigator to be related to study drug (Table 5). Any-grade TEAEs (≥ 25%) are shown in Fig. 4. The most frequent grade ≥ 3 TEAEs (≥ 15%) were neutropenia (58%) and leukopenia (36%). TEAEs led to SG dose reduction in 28% of patients, and temporary treatment interruption in 75% of patients (Table 5). A majority of the TEAEs that led to SG dose reduction and treatment interruption were hematological toxicities including neutropenia and febrile neutropenia, leukopenia, and anemia; with neutropenia being the most common TEAE leading to dose reduction (25%) and treatment interruption (61%). No TEAEs led to treatment discontinuation or death (Table 5). G-CSF was administered in a total of 39% of patients, as prophylaxis in 19% and/or as a treatment in 25% of patients. All patients received a two- or three-drug antiemetic/antinauseant regimen, which included a neurokinin-receptor antagonist in 36% of patients. The safety profile was generally similar based on *UGT1A1* genotype (Supplementary Table 2; online only).

**Table 5:** Safety summary in the phase 2 dose-expansion mTNBC cohort

Safety-evaluable patients,^a^ *n* (%)	Phase 2 mTNBC cohort (*N* = 36)
Any-grade TEAEs	36 (100)
Related to study treatment	35 (97)
Grade ≥ 3 TEAEs	26 (72)
Related to study treatment	26 (72)
Serious TEAEs	5 (14)
Related to study treatment	1 (3)
TEAEs leading to temporary treatment interruption	27 (75)
TEAEs leading to dose reduction	10 (28)
TEAEs leading to treatment discontinuation	0
TEAEs leading to death	0

*AE* adverse event, *mTNBC* metastatic triple-negative breast cancer, *TEAE* treatment-emergent adverse event

^a^TEAEs defined as any AEs that begin on or after the start of study drug through 30 days after the last dose of study drug or initiation of subsequent anti-cancer therapy, whichever occurs first

## Discussion

The open-label ASCENT-J02 phase 1/2 study, conducted to determine the RP2D of SG in Japanese patients with advanced solid tumors and to evaluate the efficacy and safety of SG in a cohort of Japanese patients with mTNBC, is the first study of SG in Japanese patients with solid tumors. In the primary analysis of phase 1, no DLTs were observed at SG 6 mg/kg and one DLT was observed at SG 10 mg/kg. Per the recommendation from the DMC, the RP2D of SG was established at 10 mg/kg, regardless of *UGT1A1* genotype. AEs observed with SG in the phase 1 part of the study, including neutropenia and diarrhea, were manageable. SG tolerability appeared to be consistent with the known safety profile of SG, with no new safety signals identified.

In the primary analysis of the phase 2 mTNBC cohort, after a median follow-up of 6.1 months (range 2.4–9.1), the confirmed ORR by IRC was 25% (95% CI 12.1–42.2). Overall, there was good concordance between the efficacy outcomes by IRC assessment and investigator assessment. Median OS had not been reached at the time of the primary analysis. While acknowledging the limits of cross-trial comparisons, and despite the shorter follow-up duration compared with ASCENT, median PFS, DOR, and TTR in this study in Japanese patients were comparable and consistent with those observed in the primary results of the global phase 3 ASCENT study. For context, among the full population of patients treated with SG in ASCENT (including those with brain metastases), after a median survival follow-up of 10.6 months, median PFS was 4.8 months (95% CI 4.1–5.8), median DOR was 6.3 months (95% CI 5.5–9.0), median TTR was 1.5 months (range 0.7–10.6), and the percentage of patients with an objective response was 31% [18]. The ORR observed in this study (25%) was slightly lower than ASCENT, however, it may have been affected by the short follow-up time, as delayed responders were not captured in this analysis.

No new safety signals were observed in the phase 2 mTNBC cohort. There were no deaths or treatment discontinuations due to TEAEs, and the proportion of patients with TEAEs leading to SG dose reductions in this study (28%) was comparable to that observed in the ASCENT study (22%). Temporary treatment interruption of SG was observed more frequently in this study compared to ASCENT (75% vs 61%, respectively); however, the sample size in this study was small and G-CSF was more widely used in ASCENT compared with this study [28]. While the rates of neutropenia and leukopenia (including any grade and grade ≥ 3) were numerically higher than in ASCENT, the rates of treatment-related grade ≥ 3 events in this study (72%) were similar to ASCENT (65%, data on file). Overall, the TEAEs observed with SG were manageable with dose modifications and/or supportive care including G-CSF treatment.

The limitations of this study included the small sample size and lack of a control arm; however, this was designed as a phase 1/2 bridging study. Data from longer term follow-up will be informative as some endpoints were not fully mature at the time of this analysis. Nevertheless, the findings in this Japanese population were consistent with the findings of the larger, global phase 3 ASCENT study.

In conclusion, in this phase 1/2 study of SG in Japanese patients with solid tumors, the RP2D of SG was established as 10 mg/kg. At the RP2D, SG demonstrated efficacy in Japanese patients with mTNBC with an acceptable safety profile. Results from the ongoing HR + /HER2 − mBC and mUC cohorts of ASCENT-J02 are pending. The results observed in this study, in conjunction with those from the global ASCENT study, support the use of SG as a new standard of care for patients with previously treated mTNBC in Japan.

## Supplementary Information

Below is the link to the electronic supplementary material.

Supplementary file1 (DOCX 89 KB)

## Data Availability

Gilead Sciences shares anonymized individual patient data upon request or as required by law or regulation with qualified external researchers based on submitted curriculum vitae and reflecting non conflict of interest. The request proposal must also include a statistician. Approval of such requests is at Gilead Science’s discretion and is dependent on the nature of the request, the merit of the research proposed, the availability of the data, and the intended use of the data. Data requests should be sent to datarequest@gilead.com.

## References

[1] Won KA, Spruck C (2020) Triple-negative breast cancer therapy: current and future perspectives (review). Int J Oncol 57(6):1245–1261 33174058 10.3892/ijo.2020.5135 PMC7646583

[2] Allison KH, Hammond MEH, Dowsett M et al (2020) Estrogen and progesterone receptor testing in breast cancer: ASCO/CAP Guideline Update. J Clin Oncol 38(12):1346–1366 31928404 10.1200/JCO.19.02309

[3] Wolff AC, Somerfield MR, Dowsett M et al (2023) Human epidermal growth factor receptor 2 testing in breast cancer: ASCO-College of American Pathologists Guideline Update. J Clin Oncol 41(22):3867–3872 37284804 10.1200/JCO.22.02864

[4] Foundation for Promotion of Cancer Research (2023) Cancer Statistics in Japan - 2023. Available at: https://ganjoho.jp/public/qa_links/report/statistics/pdf/cancer_statistics_2023.pdf. Accessed 19 Oct 2023

[5] Iwase H, Kurebayashi J, Tsuda H et al (2010) Clinicopathological analyses of triple negative breast cancer using surveillance data from the Registration Committee of the Japanese Breast Cancer Society. Breast Cancer 17(2):118–124 19466512 10.1007/s12282-009-0113-0

[6] Li CH, Karantza V, Aktan G et al (2019) Current treatment landscape for patients with locally recurrent inoperable or metastatic triple-negative breast cancer: a systematic literature review. Breast Cancer Res 21(1):143 31842957 10.1186/s13058-019-1210-4 PMC6916124

[7] Im SA, Gennari A, Park YH et al (2023) Pan-Asian adapted ESMO Clinical Practice Guidelines for the diagnosis, staging and treatment of patients with metastatic breast cancer. ESMO Open 8(3):101541 37178669 10.1016/j.esmoop.2023.101541 PMC10186487

[8] Kaufman PA, Kwon CS, Feliciano J et al (2019) Systemic therapy in second-line metastatic triple negative breast cancer (mTNBC): a systematic literature review (SLR) and meta-analysis (MA) of efficacy. Ann Oncol 30(suppl 5):Abstract360P

[9] Japan Breast Cancer Society (2023) The Japanese Breast Cancer Society Clinical Practice Guidelines for systemic treatment of breast cancer. Available at: https://jbcs.xsrv.jp/guideline/2022/. Accessed 16 Jan 2024

[10] Brufsky A, Valero V, Tiangco B et al (2012) Second-line bevacizumab-containing therapy in patients with triple-negative breast cancer: subgroup analysis of the RIBBON-2 trial. Breast Cancer Res Treat 133(3):1067–1075 22415477 10.1007/s10549-012-2008-6

[11] Park IH, Im SA, Jung KH et al (2019) Randomized open label phase iii trial of irinotecan plus capecitabine versus capecitabine monotherapy in patients with metastatic breast cancer previously treated with anthracycline and taxane: PROCEED Trial (KCSG BR 11–01). Cancer Res Treat 51(1):43–52 29458237 10.4143/crt.2017.562 PMC6333992

[12] Perez EA, Patel T, Moreno-Aspitia A (2010) Efficacy of ixabepilone in ER/PR/HER2-negative (triple-negative) breast cancer. Breast Cancer Res Treat 121(2):261–271 20229176 10.1007/s10549-010-0824-0

[13] Twelves C, Jove M, Gombos A et al (2016) Cytotoxic chemotherapy: still the mainstay of clinical practice for all subtypes metastatic breast cancer. Crit Rev Oncol Hematol 100:74–87 26857987 10.1016/j.critrevonc.2016.01.021

[14] Goldenberg DM, Cardillo TM, Govindan SV et al (2015) Trop-2 is a novel target for solid cancer therapy with sacituzumab govitecan (IMMU-132), an antibody-drug conjugate (ADC). Oncotarget 6(26):22496–22512 26101915 10.18632/oncotarget.4318 PMC4673178

[15] Rugo HS, Bardia A, Tolaney SM et al (2020) TROPiCS-02: A phase III study investigating sacituzumab govitecan in the treatment of HR+/HER2- metastatic breast cancer. Future Oncol 16(12):705–715 32223649 10.2217/fon-2020-0163

[16] Ambrogi F, Fornili M, Boracchi P et al (2014) Trop-2 is a determinant of breast cancer survival. PLoS ONE 9(5):e96993 24824621 10.1371/journal.pone.0096993 PMC4019539

[17] Vidula N, Yau C, Rugo H (2022) Trophoblast cell surface antigen 2 gene (TACSTD2) expression in primary breast cancer. Breast Cancer Res Treat 194(3):569–575 35789445 10.1007/s10549-022-06660-x

[18] Bardia A, Hurvitz SA, Tolaney SM et al (2021) Sacituzumab govitecan in metastatic triple-negative breast cancer. N Engl J Med 384(16):1529–1541 33882206 10.1056/NEJMoa2028485

[19] BioPharma APAC (2022) Singapore (HSA) approval marks the first in a series of expected approvals of Trodelvy in Asia. Available at: https://biopharmaapac.com/news/43/1252/-singapore-hsa-approval-marks-the-first-in-a-series-of-expected-approvals-of-trodelvy-in-asia.html. Accessed 19 Oct 2023

[20] Michaleas S, Moreno Oliver A, Mueller-Berghaus J et al (2022) The European Medicines Agency review of sacituzumab govitecan for the treatment of triple-negative breast cancer. ESMO Open 7(3):100497 35642987 10.1016/j.esmoop.2022.100497 PMC9149193

[21] Korea Biomedical Review (2023) Gilead's trodelvy approved for metastatic triple-negative breast cancer in Korea. Available at: https://www.koreabiomed.com/news/articleView.html?idxno=21060. Accessed 19 Oct 2023

[22] Pharmaceutical Technology (2022) Everest’s trodelvy receives approval for breast cancer in China. Available at: https://www.pharmaceutical-technology.com/news/everest-trodelvy-breast-cancer/. Accessed 19 Oct 2023

[23] TRODELVY® (sacituzumab govitecan-hziy) (2023) [prescribing information]. Gilead Sciences, Inc., Foster City, CA

[24] Europeans Medicines Agency (2021) Trodelvy [summary of product characteristics]. Available at: https://www.ema.europa.eu/en/documents/product-information/trodelvy-epar-product-information_en.pdf. Accessed 19 Oct 2023

[25] Rugo HS, Bardia A, Marme F et al (2022) Sacituzumab govitecan in hormone receptor-positive/human epidermal growth factor receptor 2-negative metastatic breast cancer. J Clin Oncol 40(29):3365–3376 36027558 10.1200/JCO.22.01002

[26] Tagawa ST, Balar AV, Petrylak DP et al (2021) TROPHY-U-01: A phase II open-label study of sacituzumab govitecan in patients with metastatic urothelial carcinoma progressing after platinum-based chemotherapy and checkpoint inhibitors. J Clin Oncol 39(22):2474–2485 33929895 10.1200/JCO.20.03489 PMC8315301

[27] Eisenhauer EA, Therasse P, Bogaerts J et al (2009) New response evaluation criteria in solid tumours: revised RECIST guideline (version 1.1). Eur J Cancer 45(2):228–247 19097774 10.1016/j.ejca.2008.10.026

[28] Rugo HS, Tolaney SM, Loirat D et al (2022) Safety analyses from the phase 3 ASCENT trial of sacituzumab govitecan in metastatic triple-negative breast cancer. NPJ Breast Cancer 8(1):98 36038616 10.1038/s41523-022-00467-1 PMC9424318

