# Supplementary material for: Preliminary results from ASCENT-J02: a phase 1/2 study of sacituzumab govitecan in Japanese patients with advanced solid tumors
Source: Int J Clin Oncol. 2024 Sep 20;29(11):1684–95. doi: 10.1007/s10147-024-02589-x (PMC11511732; doi:10.1007/s10147-024-02589-x)
Supplement: Supplementary file 1 — Supplementary file1 (DOCX 89 KB) [file 10147_2024_2589_MOESM1_ESM.docx]

# Supplementary Appendix

This appendix has been provided by the authors to give readers additional information about their work. Supplement to: Naito Y, et al. Preliminary results from ASCENT-J02: a phase 1/2 study of sacituzumab govitecan in Japanese patients with advanced solid tumors.

**Supplementary Table 1.** Safety summary by *UGT1A1* status in phase 1 dose-escalation cohorts

| **Safety-evaluable patients,^a^**  ***n* (%)** | **SG**  **6 mg/kg** | | | **SG**  **10 mg/kg** | | |
| --- | --- | --- | --- | --- | --- | --- |
|  | *UGT1A1* wild type  (n = 2) | *UGT1A1* polymorphism  (n = 4) | Total  (n = 6) | *UGT1A1* wild type  (n = 6) | *UGT1A1* polymorphism  (n = 3) | Total  (n = 9) |
| Any-grade TEAEs | 2 (100) | 4 (100) | 6 (100) | 6 (100) | 3 (100) | 9 (100) |
| Related to study treatment | 2 (100) | 4 (100) | 6 (100) | 6 (100) | 3 (100) | 9 (100) |
| Grade ≥ 3 TEAEs | 0 | 1 (25) | 1 (17) | 5 (83) | 2 (67) | 7 (78) |
| Related to study treatment | 0 | 1 (25) | 1 (17) | 5 (83) | 2 (67) | 7 (78) |
| Serious TEAEs | 0 | 0 | 0 | 1 (17) | 0 | 1 (11) |
| Related to study treatment | 0 | 0 | 0 | 0 | 0 | 0 |
| TEAEs leading to temporary treatment interruption | 1 (50) | 2 (50) | 3 (50) | 5 (83) | 2 (67) | 7 (78) |
| TEAEs leading to dose reduction | 0 | 1 (25) | 1 (17) | 2 (33) | 0 | 2 (22) |
| TEAEs leading to treatment discontinuation | 0 | 0 | 0 | 1 (17) | 0 | 1 (11) |
| TEAEs leading to death | 0 | 0 | 0 | 0 | 0 | 0 |

^a^TEAEs defined as any AEs that begin on or after the start of study drug through 30 days after the last dose of study drug or initiation of subsequent anti-cancer therapy, whichever occurs first.

*AE* adverse event; *SG* sacituzumab govitecan; *TEAE* treatment-emergent adverse event; *UGT1A1*, UDP glucuronosyltransferase family 1 member A1.

**Supplementary Table 2.** Safety summary by *UGT1A1* status in the phase 2 dose expansion mTNBC cohort

| **Safety-evaluable patients,^a^ *n* (%)** | *UGT1A1* wild type  (n = 32) | *UGT1A1* polymorphism  (n = 4) | Total  (N = 36) |
| --- | --- | --- | --- |
| Any-grade TEAEs | 32 (100) | 4 (100) | 36 (100) |
| Related to study treatment | 31 (97) | 4 (100) | 35 (97) |
| Grade ≥ 3 TEAEs | 22 (69) | 4 (100) | 26 (72) |
| Related to study treatment | 22 (69) | 4 (100) | 26 (72) |
| Serious TEAEs | 4 (13) | 1 (25) | 5 (14) |
| Related to study treatment | 1 (3) | 0 | 1 (3) |
| TEAEs leading to temporary treatment interruption | 24 (75) | 3 (75) | 27 (75) |
| TEAEs leading to dose reduction | 10 (31) | 0 | 10 (28) |
| TEAEs leading to treatment discontinuation | 0 | 0 | 0 |
| TEAEs leading to death | 0 | 0 | 0 |

^a^TEAEs defined as any AEs that begin on or after the start of study drug through 30 days after the last dose of study drug or initiation of subsequent anti-cancer therapy, whichever occurs first.

*AE* adverse event; *mTNBC* metastatic triple-negative breast cancer; *TEAE* treatment-emergent adverse event; *UGT1A1*, UDP glucuronosyltransferase family 1 member A1.

**Supplementary Fig. 1** Kaplan–Meier estimate of OS in the phase 2 dose-expansion mTNBC cohort of ASCENT-J02.


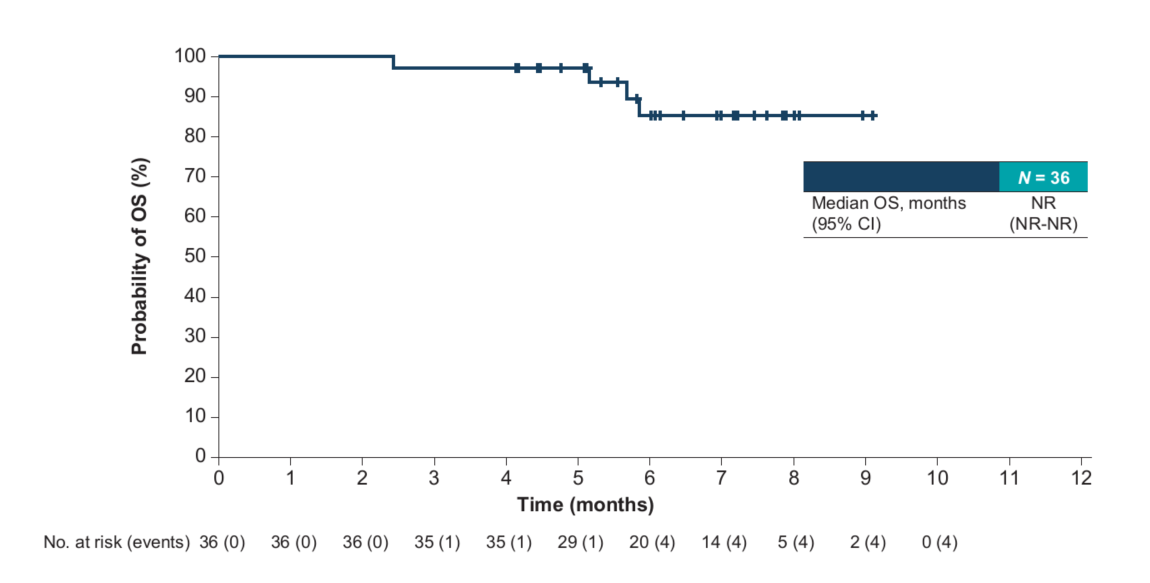


Median follow-up for OS was 6.1 months, the primary analysis for efficacy was performed ~18 weeks after the last patient enrolled.

*CI* confidence interval; *mTNBC* metastatic triple-negative breast cancer; *NR* not reached; *OS* overall survival.
